# Relationship Between Shyness and Generalized Pathological Internet Use Among Chinese School Students: The Serial Mediating Roles of Loneliness, Depression, and Self-Esteem

**DOI:** 10.3389/fpsyg.2018.01822

**Published:** 2018-10-29

**Authors:** Fengqiang Gao, Zongxin Guo, Yu Tian, Yingdong Si, Peng Wang

**Affiliations:** Department of Psychology, Shandong Normal University, Jinan, China

**Keywords:** shyness, self-esteem, loneliness, depression, GPIU, multiple mediating effects

## Abstract

The present study aimed to explore the mediating effects of loneliness, depression, and self-esteem on the association between shyness and generalized pathological Internet use (GPIU). A total of 5215 school students completed questionnaires regarding shyness, loneliness, depression, self-esteem, and GPIU (aged 11–23 years old, *M* = 16.19, *SD* = 3.10). The self-reported scores for GPIU, shyness, loneliness, depression, and self-esteem were tested in students from elementary schools to universities. The results of a variance analysis indicated that senior high school students had the greatest prevalence of GPIU of all the study stages. With the study stages resolved, the results of a structural equation model revealed that: (a) shyness positively predicted GPIU; (b) shyness/loneliness/depression predicted GPIU through self-esteem; (c) shyness predicted GPIU through loneliness/depression → self-esteem; and (d) shyness predicted GPIU through loneliness → depression → self-esteem. In conclusion, these results provided significant implications for preventing or reducing GPIU in Chinese school students.

## Introduction

### GPIU

The Internet has become increasingly popular among the Chinese population. And the 41st Statistical Report on Internet Development in China (China Internet Network Information Center [CNNIC], 2018) found that there were 772 million Internet users in China. Moderate Internet usage enables many conveniences, such as obtaining useful information, making friends (Schimmenti and Caretti, 2010; Rachmayani, 2017), and online shopping (Young and Case, 2004; Ioannidis et al., 2018). Excessive and uncontrolled use of the Internet may result in pathological Internet use (PIU), which means people are unable to control their Internet usage (Spada, 2014; Musetti et al., 2016; Kardefelt-Winther et al., 2017; Musetti and Corsano, 2018; Starcevic et al., 2018). Davis proposed a theory about PIU called the cognitive-behavioral model. It categorizes PIU as generalized pathological Internet use (GPIU) and specific pathological Internet use (SPIU) (Davis, 2001). GPIU was used in this study. Previous study showed that school students are the largest population of Internet users and the GPIU phenomenon is also very prevalent (China Internet Network Information Center [CNNIC], 2018). Studies have repeatedly found that GPIU has many consequences on school students (Shi et al., 2017; Geng et al., 2018; Marengo et al., 2018). For example, GPIU may lead to poor academic performance and social difficulties (Cao et al., 2018; Settanni et al., 2018), negative emotions (such as depression and loneliness) (Schimmenti et al., 2014; Sharifpoor et al., 2017; Zhao H. et al., 2017; Marengo et al., 2018; Settanni et al., 2018), lower self-esteem (Seabra et al., 2017), less sleep, poor eating patterns (Fuchs et al., 2018), body image dissatisfaction and internalizing symptoms (Schimmenti et al., 2014; Marengo et al., 2018), decreased visual function (Rachmayani, 2017), and the emergence of psychiatric disorders (Fuchs et al., 2018). Thus, identifying the antecedent variables of school students’ GPIU and designing effective interventions are urgent.

### Shyness and GPIU

Previous studies showed that people with shyness often feel uncomfortable when they interacted with strangers (Cheek and Buss, 1981). And they tend to spend more time on the Internet (Saunders and Chester, 2008; Eldeleklioglu and Vural-Batik, 2013; Li et al., 2016; Ang et al., 2017; Tian et al., 2017). There are several reasons why shy individuals easily develop GPIU. First, social anxiety is a core trait of shy individuals; it causes them discomfort and inhibition in response to real social interactions (McCabe, 2015). They therefore decrease offline interactions and ultimately feel a lack of social resources and support (Porter and Chambless, 2017). However, previous studies and the social compensation model have found that shy individuals tend to obtain social resources and support from online activities such as social networking or Internet gaming (Kraut et al., 2002; Jin, 2013; Li et al., 2016; Sioni et al., 2017; Miao et al., 2018). Once shy individuals obtain the social resources and support they need from the Internet, they will spend more time online (Tian et al., 2017). Second, shy individuals tend to be apprehensive of being evaluated in real life social activities, and especially fear of being disapproved when they share their inner thoughts and emotions. ACE theory (anonymity, convenience, and escape from reality) states that the Internet’s traits of anonymity and escape from reality could decrease the possibility of identification by others and make shy people feel safe to share things they cannot offline (Young et al., 2000; Schimmenti and Caretti, 2010; Barlett, 2015), which increases the possibility of online interactions with others (Jin et al., 2017). Third, shy individuals have some visibly negative symptoms (such as blushing) when they interact with others in real life, and these make shy people feel uncomfortable in face–to–face interactions (Nikolić et al., 2016). Self-presentation theory indicates that shy people may experience some uncomfortable feelings in face–to–face interactions, because they are more likely to notice hostile auditory and visual cues from others, so they prefer to use the Internet to communicate (Stritzke et al., 2004; Schimmenti et al., 2017; Settanni et al., 2018). Hence, shy individuals are apt to be online frequently and eventually develop GPIU. Exploring the potential mechanisms by which shy people easily develop GPIU is very important.

### The Mediating Roles of Self-Esteem, Loneliness, and Depression

To understand the etiology behind PIU and intervene effectively, Davis provided theoretical explanations of its origins and pathogenesis, which is called the cognitive-behavioral model (Davis, 2001). In this model, psychopathology (such as social anxiety or depression) was considered as a distal factor that will result in GPIU (Davis, 2001; Musetti et al., 2018). However, Davis indicated that even these psychopathology factors are necessary in the cognitive-behavioral model, while they are not the key factors that are in the proximal position that will inevitably lead to GPIU. The key factors and proximal causes that will result in GPIU are maladaptive cognitions. Previous studies also showed that some distal psychopathological factors could trigger an individuals’ GPIU via the proximal factor of maladaptive cognition (Kalkan, 2012; Li and Wang, 2013; Lu and Yeo, 2015; Tian et al., 2017).

Based on Davis’ cognitive-behavioral theory and previous studies, we considered personalities of shyness, loneliness, depression as the distal psychopathology factors that will result in GPIU through maladaptive cognition (Davis, 2001; Goossens et al., 2009; Majorano et al., 2015; Corsano et al., 2017). Shyness tend to be a distal psychopathology factor because social anxiety is its core trait (Hofmann et al., 2006; McCabe, 2015). One reason people experience loneliness is because they feel anxiety when interacting with other people; they are likely to decrease interactions with others until they eventually experience more loneliness (Fang and Sun, 2018). Therefore, shyness, loneliness, and depression tend to be the distal factors that make individuals very vulnerable to GPIU. According to cognitive-behavioral theory, maladaptive cognition is the key and proximal factor that leads to GPIU (Davis, 2001). Previous studies investigated cognitive factors that caused GPIU and found that one was self-esteem. Self-esteem is defined as an evaluation of self-concept or how people think about themselves, which was deemed as a crucial antecedent for GPIU (Davis, 2001; Shi et al., 2017). Previous studies also showed that low self-esteem is a maladaptive cognition that can result in many negative consequences such as GPIU (Davis, 2001; Shi et al., 2017).

Previous studies found that shyness tended to be the antecedent variable for loneliness and depression (Jackson et al., 2002; Mahon et al., 2006; Zhao et al., 2012, 2013). First, the cognitive bias model indicates that there is a negative cognitive process from some individual difference variables (such as shyness) to loneliness (Levin and Stokes, 1986; Zhao et al., 2018). This may be because shy individuals are afraid of the negative opinions of others, which may make them feel more likely to decrease social interactions, which eventually experience greater loneliness. Many studies have found similarities between loneliness and depression, since some report depression has components of loneliness (Musetti et al., 2018; Zhao et al., 2018). We therefore consider shyness as an effective predictor of both loneliness and depression. Second, social anxiety is the core trait of shyness (Snyder et al., 1985; McCabe, 2015). Previous studies indicated that people with social anxiety fear interacting with others and have poor interpersonal relationships (Capriola-Hall et al., 2018). This paucity of interactions with others and undesirable interpersonal relationships are the important influential factors of loneliness and depression.

Depression is a syndrome with symptoms such as feelings of worthlessness, emotional collapse, pessimism, indifference, and deep sorrow (McKenzie et al., 2010). Many factors are the influential factors of depression, including loneliness (Vanhalst et al., 2012; Majorano et al., 2015; Musetti et al., 2018). A possible reason is that young adults tend to cope with loneliness using maladaptive cognitions and strategies such as blaming themselves and engaging in passive coping strategies instead of active ones (Cacioppo and Hawkley, 2009). These maladaptive strategies further enhance their feelings of loneliness. Extreme and ongoing loneliness may stimulate depressive personality trait, which is an asymptomatic trait. There is a well-documented strong association between loneliness and depression; a meta-analysis conducted in 2006 analyzed the findings of 33 studies to confirm this association (Mahon et al., 2006).

### School Students and the Internet

Students were selected as the sample in this study for several reasons. First, school students face significant academic pressures, and engaging in Internet activities (such as online gaming) can help them relieve pressure and feel better emotionally (Dutta and Chye, 2017; Longobardi et al., 2018; Settanni et al., 2018). Therefore, students who use the Internet frequently may develop GPIU. Second, most students must focus on their studies and therefore have limited time and money to purchase products (Li, 2017). However, online shopping can be a very convenient and economical way to buy goods (Zhao H. et al., 2017). Hence, students use the Internet more frequently. Third, Chinese school students are nested in fixed classrooms where the rules are strictly enforced by their teachers and parents. Therefore, peer pressure and herd behavior more readily occur in China than in Western countries. Thus, students who do not use the Internet may be easily influenced by those who frequently use the Internet (Li and Zeng, 2017). Fourth, many school students are interested in pursuing new and varied experiences. The Internet can bring them positive short-term effects on mood and satisfy their demands for convenience, speed, and novelty (such as Internet gaming and 3D movies) (Chao and Yu, 2017; Settanni et al., 2018). Therefore, students are attracted to the Internet and spend more time online. We can conclude from the aforementioned discussions that Internet use among school students is very prevalent; this is the main reason we used them as our sample.

### Study Stage Differences of GPIU

Understanding the prevalence of Internet use among school students, we hypothesize that their GPIU behavior may differ in varying study stages. There are many differences in family and school environments between the four stages of this study. First, elementary and junior high school students spend a lot of time in the company of family members, and in school environment. Their interactions with their parents and teachers are closer than those of senior high school and university students, and the supervision and guidance of parents and teachers is very strict (Azmitia et al., 2009). Most elementary and junior high school students do not have Internet access at school and their parents tend to limit their online time at home. Most senior high school and university students spend a great deal of time on campus and have fewer interactions with their parents and teachers. The lack of strict supervision from parents and teachers allows them more free time to use the Internet (Tian et al., 2017). Therefore, senior high school and university students may use the Internet a lot, and may more easily develop GPIU than elementary and junior high school students. Second, the students in the different study stages may prefer specific types of Internet use. Previous studies indicated that elementary and junior high school students prefer to play online games when they use the Internet. Senior high school and university students not only play Internet games, but also frequently use social networking sites. This may be because senior high school and university students have more interpersonal relationship problems and feel greater loneliness than students in the other stages. The Internet allows them to satisfy their emotional needs and ease their loneliness through social networking (Brand et al., 2014). Third, elementary school and university school students have less educational pressure and therefore may have more free time to use the Internet. Junior and senior high school students have many examinations and heavy academic workloads, which may bring them great pressure and limits their time for Internet use (Ishige and Muto, 2005). However, junior and senior school students with unsatisfactory academic performances may feel hopeless and experience many negative emotions such as loneliness and anxiety. Using the Internet helps relieve those pressures and negative emotions, prompting them to use the Internet more frequently, which may finally lead to the development of GPIU.

Based on the aforementioned discussions, the following hypotheses were proposed:

**Hypothesis 1.** Shyness can positively predict GPIU.
**Hypothesis 2.** Shyness/loneliness/depression can predict GPIU through self-esteem.
**Hypothesis 3.** Shyness can predict GPIU through loneliness/depression → self-esteem.
**Hypothesis 4.** Shyness can predict GPIU through loneliness → depression → self-esteem.
**Hypothesis 5.** Study stage differences exist for GPIU.

## Materials and Methods

The present study was performed in conformity to the code of ethics of the World Medical Association (Declaration of Helsinki) for experiments involving humans and was approved by the Ethics Committee of Shandong Normal University. Besides, and our research was obtained the written informed consent from the parents of the participants.

### Participants

The participants of this research were from three cities in Northeastern China, including two universities, an elementary school, and a junior and senior high school. Students in the first through fifth grades were not included because they did not have a QQ or WeChat number, which are the most popular instant messaging platforms in China. Another reason was their poor understanding of the scales we used. A total of 5500 school students participated in this study; 285 were eventually excluded due to incomplete questionnaires. A total of 5215 valid questionnaires were received. Overall, 2303 (44.16%) of the participants were male and 2827 (54.21%) were female. Their mean age was 16.19 years (aged 11–23 years old, *M* = 16.19, *SD* = 3.10). More specifically, 546 (aged 11–14 years old, *M* = 11.59, *SD* = 0.6; 264 male) are elementary school students, 1710 (aged 11–16 years old, *M* = 13.5, *SD* = 0.99; 822 male) are junior high school students, 688 (aged 14–18 years old, *M* = 16.22, *SD* = 1.01; 303 male) are senior high school students, 2271 (aged 15–23 years old, *M* = 19.25, *SD* = 1.74; 914 male) are university students.

### Procedures

We obtained informed consent from the school administrators and students before data collection. To maintain the quality of the investigation, the university students were gathered in a large assembly room to complete their questionnaires with the help of two researchers. The other grades finished the questionnaires with the help of two researchers during one full class period of 45 min.

### Measures

#### The Revised Henderson Undergraduate Shyness Scale (RHUSS)

The RHUSS was adapted from the Henderson and Zimbardo Shyness Scale (Henderson and Zimbardo, 2002). And it has 17 items and students rated on a 5-point scale (1 = strongly disagree to 5 = strongly agree). The final RHUSS score was calculated by the total score of all the items, with higher scores indicating severer shyness. We also conducted a confirmatory factor analysis (CFA), with *χ^2^/df* = 3.944, *p* < 0.001, RMSEA = 0.024, CFI = 0.992, IFI = 0.992, AGFI = 0.986 and GFI = 0.994. Cronbach’s alpha, composite reliability (CR) and average variances extracted (AVE) for the RHUSS were 0.869, 0.803, and 0.329.

#### Revised Rosenberg Self-Esteem Scale

The Chinese school students’ self-esteem was assessed by Revised Rosenberg Self-Esteem Scale. The scale was adapted to the Chinese language and culture. It consists of seven items. One sample item is: “I am able to do things as well as most other people.” The students rated each item on a 5-point scale (1 = strongly disagree to 5 = strongly agree). The final score was calculated by the total score of all the items, with higher scores mean higher self-esteem. We also conducted a CFA, with *χ^2^/df* = 6.440, *p* < 0.01, RMSEA = 0.032, CFI = 0.999, IFI = 0.999, AGFI = 0.990, and GFI = 0.999. Cronbach’s alpha, CR and AVE for the Revised Rosenberg Self-Esteem Scale were 0.816, 0.869, and 0.500.

#### The UCLA Loneliness Scale

The UCLA Loneliness Scale consists of 20 items. One sample item is: “I am unhappy doing so many things alone” (Russell et al., 1978). The items were rated on a 4-point scale for frequency (1 = often to 4 = never). The final score was calculated by the total score of all the items, with higher scores indicating greater levels of loneliness. We also conducted a CFA, with *χ^2^/df* = 3.846, *p* < 0.01, RMSEA = 0.023, CFI = 0.993, IFI = 0.993, AGFI = 0.985, and GFI = 0.993. Cronbach’s alpha, CR and AVE for the UCLA Loneliness Scale were 0.855, 0.779, and 0.284.

#### Centre for Epidemiologic Studies Depression Scale (CES-D)

The CES-D Scale was used to measure school students’ depression, which was adapted to the Chinese language and culture. It consists of 20 items. One sample item is: “I felt that I could not shake off the blues even with help from my family or friends.” The students rated each item on a 4-point scale varying from 1 = rarely or none of the time (less than 1 day) to 4 = most or all of the time (5–7 days). To calculate the final CES-D score, the scores of all the items were added up. A very high score was taken to mean severe depression. We also conducted a CFA, with *χ^2^/df* = 3.510, *p* < 0.001, RMSEA = 0.022, CFI = 0.994, IFI = 0.994, AGFI = 0.986, and GFI = 0.993. Cronbach’s alpha, CR and AVE for the CES-D were 0.894, 0.837, and 0.362.

#### Generalized Pathological Internet Use Scale (GPIUS)

Patricia Gomez’s GPIUS was adapted and developed to measure the Chinese students’ generalized pathological use of the Internet (Gómez et al., 2017). The GPIUS consists of 11 items. One sample item is: “You have connected to the Internet even though you knew it could get you in trouble.” The GPIUS were rated on a 7-point Likert scale (1 = completely disagree to 7 = completely agree). The final GPIUS score was calculated by the total score of all the items, with higher scores indicating severer GPIU. We also conducted a CFA, with *χ^2^/df* = 3.794, *p* < 0.001, RMSEA = 0.023, CFI = 0.998, IFI = 0.998, AGFI = 0.991, and GFI = 0.998. Cronbach’s alpha, CR and AVE for the GPIUS were 0.883, 0.906, and 0.472.

Additionally, though the indexes of Cronbach’s alpha, CR of these instruments were within the acceptable range. However, most of the instruments have a low average variance extracted (less than 0.50) and these instruments have to be purified and improved so that they acquire adequate psychometric properties.

### Statistical Analysis

To test the common method biases, the common variance analysis was conducted by the factor analysis. Then we analyzed the statistics via descriptive and correlation analyses. The third step evaluated the multiple mediation. We finally used a structure equation model to evaluate a multiple mediation model for the roles of loneliness, depression, and self-esteem in the relationship between shyness and GPIU.

In addition, we use the parceling strategy to enhance the quality of the model fit and the indicators. A factor analysis was firstly executed, and the items of each observed variable were ranked from the highest to the lowest on the basis of the factor loading size (Rogers and Schmitt, 2004). Each parcel was sequentially assigned the remaining items with the highest and lowest rankings, alternating via the parcels, until all of the items were finished.

The fits of the model were assessed using the chi-squared (*χ^2^*) test, the root mean square error of approximation (RMSEA), the comparative fit index (CFI), and the Tucker-Lewis index (TLI). Considering the chi-squared test is easily affected by the size of samples, model fit indices were used as the major standard to assess the model fit. The CFI and TLI ranged from 0 to 1, with values above 0.90 representing sufficient model fit (Hu and Bentler, 1999). A criterion of thumb for the RMSEA is that values ≥0.10 represent poor fit, values between 0.05 and 0.08 represent reasonable error of approximation, and values ≤0.05 represent close approximation (Browne and Cudeck, 1992). The fits of this model were *χ^2^/df* = 3.351, RMSEA = 0.021, NFI = 0.997, CFI = 0.998, and TLI = 0.995, which indicated that this was an appropriate and acceptable model.

The bootstrapping method was conducted to test the mediation effects. This method produced 95% bias-corrected confidence intervals of these effects from 1000 resamples of the data. Confidence intervals that did not contain zero indicated effects that were significant at *α* = 0.05. We finally executed a path analysis, a structural equation model (SEM) for the observed variables, to further validate our theoretical model.

## Results

### Common Method Biases

The common variance analysis was conducted to measure if common method biases existed in the present study. The chi-squared test of Bartlett’s test of sphericity was significant. After a principal component analysis, 12 eigenvalues greater than 1 were extracted. The first factor to explain the variance was 20.565%. And the results was less than the critical standard of 40% (Podsakoff et al., 2003), indicating that these instruments had no problem with the common method biases.

### Descriptive Statistics and Correlation Analysis

Table 1 showed the descriptive statistics and Pearson correlations of the study stages, shyness, loneliness, self-esteem, depression and GPIU. The results indicated that shyness, loneliness, depression and GPIU were positively associated with one another (*p* < 0.01) and were negatively associated with self-esteem (*p* < 0.01).

**Table 1:** Descriptive statistics and correlation matrix of all variables.

Variables	*M*	*SD*	1	2	3	4	5
1.Study stage	2.90	1.08	–				
2.Shyness	46.30	11.51	0.067ˆ**				
3.Self-esteem	36.64	7.33	0.122ˆ**	-0.303ˆ**			
4.Loneliness	41.81	9.09	0.025	0.448ˆ**	-0.517ˆ**		
5.Depression	17.71	10.24	0.006	0.456ˆ**	-0.541ˆ**	0.636ˆ**	
6.GPIU	37.25	13.28	0.192ˆ**	0.371ˆ**	-0.195ˆ**	0.284ˆ**	0.367ˆ**

*Note: *N* = 5215. ^∗∗^*p* < 0.01*.

### Study Stage Differences of the Variables

The study stage differences of shyness, loneliness, self-esteem, depression, and GPIU are provided in Table 2. The results show that senior high school students were the most vulnerable to GPIU at any stage. After understanding how the study stage affected GPIU, it was used as the control variable in the SEM. Additionally, according to Ferguson’s (2009) suggestion, all the effect sizes are small, which means the study stage differences of shyness, loneliness, self-esteem, depression, and GPIU should be explained with caution.

**Table 2:** Study stage differences of variables.

	Elementary		Junior school		Senior school		University				The order of different study stage of each variable
	*M*	*SD*	*M*	*SD*	*M*	*SD*	*M*	*SD*	*F*	*η*^2^	
Shyness	45.56	10.04	45.04	11.49	48.01	13.52	46.91	11.08	14.92ˆ***	0.009	M_Senior_ > M_University_ > M_Elementary_ > M_Junior_
Self-esteem	35.47	7.57	35.82	7.74	36.12	7.74	37.70	6.67	29.01ˆ***	0.016	M_University_ > M_Senior_ > M_Junio_ > M_Elementary_
Loneliness	41.11	9.26	41.61	9.12	42.62	10.24	41.88	8.64	3.24ˆ*	0.002	M_Senior_ > M_University_ > M_Junio_ > M_Elementary_
Depression	16.37	10.00	17.84	10.24	19.72	11.14	17.33	9.94	13.21ˆ***	0.008	M_Senior_ > M_Junio_ > M_University_ > M_Elementary_
GPIU	28.41	12.65	36.79	13.67	39.70	14.01	38.99	11.94	108.48ˆ***	0.059	M_Senior_ > M_University_ > M_Junio_ > M_Elementary_

*Note: *N* = 5215. ^∗^*p* < 0.05, ^∗∗∗^*p* < 0.001. Effect size interpretation suggestions: *η*^*2*^ = 0.04 minimum; *η*^*2*^ = 0.25 moderate; *η*^*2*^ = 0.64 strong (Ferguson, 2009). *M* = Mean*.

### Testing for Multiple Mediating Roles of Self-Esteem, Loneliness and Depression

**FIGURE 1:**
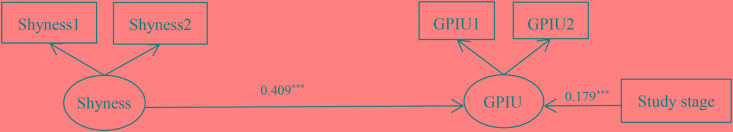
Total effect model. Path values are the path coefficients. ^∗∗∗^*p* < 0.001.

First, the SEM was used to examine direct effect from shyness to GPIU. It found that shyness positively predicted GPIU (see Figure 1).

**FIGURE 2:**
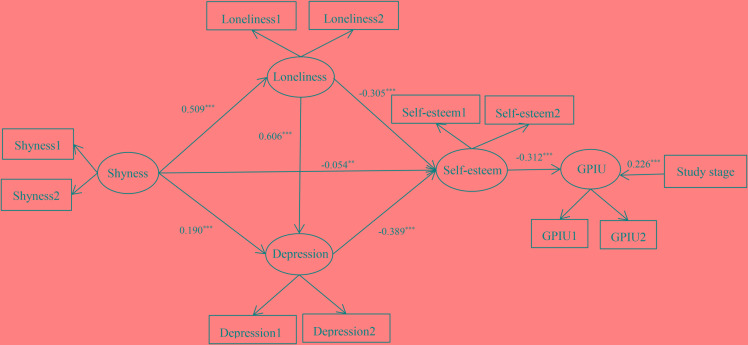
Multiple mediation model. ^∗∗^*p* < 0.01, ^∗∗∗^*p* < 0.001.

Second, Mplus 7.0 was used to explore the indirect effects of loneliness, depression and self-esteem between shyness and GPIU, and the results supported both of our hypotheses (see Figure 2). Specifically, shyness positively predicted loneliness and depression but negatively predicted self-esteem. Likewise, loneliness positively predicted depression, whereas both loneliness and depression negatively predicted self-esteem. Additionally, self-esteem negatively predicted GPIU.

Third, bootstrapping was used to examine the mediated effects of loneliness, depression, and self-esteem between shyness and GPIU. All of the mediated effects paths were significant in this study (see Table 3). Specifically, self-esteem mediated the link between shyness/loneliness/depression and GPIU. Shyness predicted GPIU though loneliness/depression → self-esteem and shyness predicted GPIU though loneliness → depression → self-esteem.

**Table 3:** Testing the total effect model and the mediation effect model.

Path	Standardized path coefficient	95% confidence interval	
		Lower	Upper
**a. Total effect model**			
Shyness→GPIU	0.126^∗∗∗^	0.109	0.141
**b. Multiple mediation model**			
Indirect effects			
Shyness→self- esteem→GPIU	0.017^a^	0.008	0.026
Shyness→loneliness→self- esteem→GPIU	0.048^a^	0.041	0.056
Shyness→depression→self- esteem→GPIU	0.023^a^	0.019	0.027
Shyness→loneliness→ depression→self- esteem→GPIU	0.037^a^	0.032	0.043

*Note: *N* = 5215. ^∗∗∗^*p* < 0.001*. *^*a*^Empirical 95% confidence interval does not overlap with zero*.

## Discussion

Since the potential mechanisms by which shyness affects GPIU were unclear, this study further explored the association between shyness and GPIU with the mediating roles of loneliness, depression, and self-esteem by referencing the cognitive-behavioral model of PIU. Correlation analysis showed the associations between loneliness, depression, shyness, self-esteem, and GPIU were significant, which was consistent with the study’s theoretical assumptions. Additionally, the results of a variance analysis of GPIU, shyness, loneliness, depression, and self-esteem indicated that senior high school students are in a special and sensitive stage to GPIU, which needs further discussion concerning the reasons and possible interventions. The total effect model indicated that shyness could positively predict GPIU, and Hypothesis 1 was supported. The multiple mediation model showed that shyness/loneliness/depression affected GPIU through self-esteem and thus supported Hypothesis 2. The model also indicated that the two mediators of loneliness and depression parallel and sequentially mediate the relationship between shyness and GPIU through self-esteem, which supports Hypotheses 3 and 4.

### Study Stage Differences of GPIU

The results of the variance analysis showed that senior high school is the most sensitive and vulnerable stage for GPIU. This may be because with the progress of puberty, senior high school students have a strong sense of independence and maturation. However, parents and teachers continue to treat them as adolescents. Because of its anonymity, the Internet satisfies students’ psychological needs (Young et al., 2000). Hence, senior high school students use the Internet more frequently and may develop GPIU. Second, Chinese senior high school students face college entrance examinations that will determine their future. This causes significant stress (Xiong et al., 2016). On the one hand, students lack sufficient social interactions because they are busy studying. They therefore feel greater loneliness and depression. Lack of communication with others also aggravates the level of shyness. However, previous studies and the social compensation model indicate that engaging in online activities (such as social networking or gaming) helps relieve negative feelings (Schimmenti and Caretti, 2010; Brand et al., 2014). Alternatively, negative emotions such as anxiety and depression can occur in students who have unsatisfactory academic performance or who feel hopeless. However, the ease and comfort of virtual reality helps them escape real life, and may lead to GPIU. From the aforementioned discussions, we can conclude that senior high school students are very vulnerable to GPIU, indicating that teachers and parents should focus more on senior high school students’ GPIU behaviors.

### The Key Mediating Role of Self-Esteem

Hypothesis 2 was also supported, which meant shyness/loneliness/depression significantly associated with self-esteem, which in turn was significantly related with GPIU. That is to say, self-esteem mediated the relationship between shyness/loneliness/depression and GPIU. The results of this study supported the cognitive-behavioral model provided by Davis. Self-esteem is a key factor leading to GPIU (Davis, 2001). Self-esteem is defined as an evaluation of self-concept or how people think about themselves (Lin et al., 2018). Many studies have focused on self-esteem. Some researchers have proposed the sociometer theory of self-esteem from the perspective of evolutionary psychology (Lin et al., 2018). This theory posits that interpersonal relationship problems, which are strongly associated with shyness, loneliness, and depression, are very detrimental to self-esteem (Bayram Özdemir et al., 2017; Lin et al., 2018; Teo et al., 2018). Those with recurrent problematic interpersonal relationships may have poor self-esteem that untreated can cause emotional and behavioral issues (Leary et al., 1995). Previous studies have indicated that GPIU could be a behavioral problem manifested via Internet (Park et al., 2014; Schimmenti et al., 2014). Hence, these results are in accordance with the sociometer theory of self-esteem, which means that self-esteem mediates the effect of shyness/loneliness/depression on GPIU.

Additionally, the conceptual framework proposed by DuBois et al. (2002) was used to examine the influence of perceived social support and self-esteem on GPIU. The results found that perceived social support are strongly associated with GPIU though the mechanism of self-esteem (Lin et al., 2018). Previous studies have indicated that those with shyness, loneliness, and depression preferred to decrease social interactions with others and always perceived a lack of social support (Zhao et al., 2013, 2018). The Internet may increase their self-esteem as it affords anonymity and permits personal interactions (Young et al., 2000). Anonymity enables those who have low self-esteem to communicate with others in a safe environment, leading to feelings of belonging to a community and social acceptability, thus enhancing their self-esteem. Some individuals feel more comfortable in the online world than the offline world, which may ultimately result in GPIU. Hence, these results are in accordance with DuBois’ theory indicating that self-esteem plays a key mediating role between shyness/loneliness/depression and GPIU.

### Mediating Roles of Loneliness and Depression

In the mediation process of shyness → loneliness/depression → self-esteem → GPIU, the results of the present research were consistent with other related research that found positive relationships between shyness and loneliness/depression (Zhao et al., 2018). A possible reason is that people who are shy often view themselves negatively and feel nervous when being evaluated. They therefore tend to have many negative thoughts and emotions and fear engaging in social activities, which may make them feel lonely and depressed (Zhao et al., 2013, 2018). Second, social anxiety is the core trait of shyness (Hofmann et al., 2006), which makes them fear of interacting with others in real-life. Insufficient social interactions will worsen the sense of loneliness and even lead to depression (Sariçam et al., 2015; Lim et al., 2016; Schimmenti and Caretti, 2017). Visible symptoms such as blushing may appear when shy people try to interact with others; these cause even more discomfort and further decrease social interactions (Nikolić et al., 2016). Finally, lack of interactions with others may cause shy individuals greater feelings of loneliness and depression. Based on the aforementioned discussion, shyness affects GPIU via loneliness/depression and self-esteem.

The present study also revealed that loneliness and depression mediate the link between shyness and GPIU through self-esteem under the sequential model. These findings also indicated that loneliness is strongly correlated with depression, which is in accordance with the results of previous researches (Mahon et al., 2006; Martin and Hartley, 2017). Although this relationship has been well-documented, few studies have attempted to further explain it or determine causality. Research conducted in 2012 found that although loneliness and depression both seem to cause one another, loneliness is the stronger causal predictor of depression, which is in accordance with the findings of the present research (Vanhalst et al., 2012). A possible explanation is that those with loneliness tend to persistently adopt a series of depressive cognitions that are related to perceived social isolation (Cacioppo and Hawkley, 2009). Chronic and significant loneliness coupled with negative perceptions may ultimately lead to depression.

## Implications for the Prevention of Gpiu

The results of the present research has implications for prevention and intervention strategies on GPIU in Chinese school students. First, the results of the variance analysis indicated that the senior high school students are very vulnerable to GPIU, because of substantial academic pressure, and being away from home. Hence, parents and teachers should focus on school students with GPIU; classes in self-control and time management should be implemented. Furthermore, to enable all students with GPIU to overcome Internet dependence, there are two kinds of intervention strategies to support students with GPIU to limit their internet usage: one is to build a stronger sense of self-esteem, and to combat feelings of loneliness and depression. In this regard, cognitive-restructuring has proved to be useful (Davis, 2001). Moreover, other interventions could focus on initial variables such as shyness, loneliness, depression, and the relationships between these factors. Previous studies found that one of the core traits of shyness is social anxiety, which inhibits social interactions and is related to loneliness, depression, and low self-esteem (Snyder et al., 1985; McCabe, 2015; Fang and Sun, 2018; Heeren et al., 2018). Thus, it would be advisable that middle and high school students participate in group counseling activities on a regular basis: to promote positive interactions, reduce loneliness and depression, and enhance self-esteem.

## Limitations and Future Directions

Although the present research made many important contributions to understanding the potential mechanism of the association between shyness and GPIU in Chinese students, it has several limitations. First, the present study was a cross-sectional study, and the results could not provide causal relationships. Future study can employ experiments and longitudinal studies to explore the causal relationships between these variables. Another limitation would be the questionnaire we used in the present study, which may lead to some errors. Therefore, future studies may apply more professional surveys (e.g., SurveyMonkey or Google Forums) which would allow for the direct recording of the time spent, eliminating errors of coding the data and controlling the answers at random. Besides, most of the instruments in the present study have a low average variance extracted (less than 0.50) and these instruments have to be purified and improved so that they acquire adequate psychometric properties. Finally, the loneliness scale used in this study might also produce some limitations because it focuses on a single dimension. However, future study can explore the relationship between multidimensional loneliness and GPIU, self-esteem and depression (Goossens et al., 2009; Corsano et al., 2017). Finally, the present research was conducted in a Chinese cultural setting and the cross-cultural applicability of the conclusions must be properly verified. Related research should be conducted in different countries and cultures.

## Conclusion

(a) The self-reported scores for GPIU and shyness, loneliness, depression, and self-esteem were tested in students from elementary schools to universities; senior high school students had the greatest prevalence of GPIU in all of the study stages.
(b) Shyness positively predicted GPIU.
(c) Shyness/loneliness/depression predicted GPIU through self-esteem.
(d) Shyness predicted GPIU through loneliness/depression → self-esteem.
(e) Shyness predicted GPIU through loneliness → depression → self-esteem.

## Author Contributions

FG and ZG contributed equally to this study and share the first authorship. YT, YS and PW are the co-corresponding authors.

## Conflict of Interest Statement

The authors declare that the research was conducted in the absence of any commercial or financial relationships that could be construed as a potential conflict of interest.
